# Bioelectrical signal processing in cardiac and neurological applications and electromyography: physiology, engineering, and noninvasive applications

**DOI:** 10.1186/1475-925X-6-27

**Published:** 2007-07-03

**Authors:** Max E Valentinuzzi

**Affiliations:** 1 Instituto Superior de Investigaciones Biológicas (INSIBIO) Universidad Nacional de Tucumán (UNT), Tucumán, Argentina; 2 Consejo Nacional de Investigaciones Científicas y Técnicas (CONICET) 4000 Tucumán, Argentina

## Abstract

The present article reviews two recent books dealing with rather closely related subjects; in fact, they tend to complement and supplement reciprocally. Obviously, the electromyogram is a bioelectrical signal that often is mathematically manipulated in different ways to better extract its information. Moreover, its correlation with other bioelectric variables may become necessary.

Civil Engineering appeared, say, during the Industrial Revolution (XVIIIth-XIXth Centuries), even though there are countless previous contributions in hydraulics, mechanics and other areas [1], making use of ingenuity in civilian applications at large, as some kind of counter-face of Military Engineering. Specializations within the former showed up slowly first (Naval, Mechanical, Electrical) to speed up its diversification in the XXth Century with Telecommunications, Electronics, Computers and several other branches and sub-branches. Biomedical Engineering/Bioengineering emerged in the 1950's as a fascinating interdisciplinary blend, where biology and medicine encompass targets that need to be better understood, in a steady and not easy quantification process. Their signals must be read and interpreted, beyond the limited abilities of the naked eye and the associated human experience [2]. Here is where the field of Biomedical Signal Processing (BSP) takes personality in its own right as interdisciplinary indispensable tool, full of ingenious new ideas and concepts.

Bioelectrical signals still continue to excite physicians and engineers alike. Processing techniques have helped uncover information which completely changed the way various diseases were previously diagnosed. The aim of the first of these books, as clearly stated by its authors,**is to present a comprehensive overview of techniques with particular relevance to the processing of these signals, mainly the electrocardiogram (ECG), the electroencephalogram (EEG), the electromyogram (EMG) and evoked potentials (EP)**. Extension to other less commonly recorded physiological events should not be difficult for the motivated and keen professional.

It is intended for final year undergraduate and graduate students in biomedical engineering, electrical engineering and computer science. It may also be used as reference for practicing engineers, physicians, researchers and anyone interested in finding out what information can be derived from bioelectrical signals. Its authors, one from Lund University, in Sweden, and the other from Zaragoza University, in Spain, are well-prepared and experienced professionals who neatly have produced an excellent and recommendable piece.

Let us now make a quick tour through the book starting with the preface (6 pp, [6] references), where the aim is stated, the content is overviewed and several acknowledgments are made, showing that a good number of quite capable people have contributed to highlight the product.

Its first chapter, the introduction (24 pp, 8 figures, 32 references,), puts BSP in context and gives a brief description of bioelectricity. The complexity of a signal is often considerable and its processing has become a convenient and efficient way for extracting clinically significant information many times hidden. A fundamental objective of BSP is to *reduce the subjectivity *of manual measurements; in addition, it may be used in its own right for developing methods to *extract features *which help characterize and understand the information carried by a signal. Another objective of BSP is *noise reduction*, whatever the origin of the interfering signal is. *Mathematical modeling *and *simulation *constitute other important objectives which can help in a better understanding of physiological phenomena. Besides, the chapter points out to three major clinical contexts for BSP, namely, *diagnosis*, *therapy *and *monitoring*; the two latter may not sometimes be obvious. Clear definitions of *sensitivity*, *specificity*, *positive *and *negative predictive values *are given while the importance of databases is underlined.

The electroencephalogram is the subject matter dealt with in chapter 2 (25 pp, 13 figures, 50 references), so providing the reader with basic general knowledge about brain activity and serving as background to the following chapter 3, on EEG signal processing (87 pp, 38 figures, 205 references, 21 problems). The latter is the true beginning of the book, as it offers the "meat and potatoes" to start chewing and tasting in a 6-section tray dealing, respectively, with modeling the EEG signal, artifacts in the EEG, nonparametric spectral analysis, model-based spectral analysis, EEG segmentation, and joint time-frequency analysis. Should the EEG be viewed as a deterministic or stochastic event? If the latter description is chosen, which type of probability density function (PDF) will provide adequate statistical characterization of the signal? Are there cases where the EEG signal should be treated as non-stationary or non-Gaussian? What are the most frequent sources of interference and what methods are best suited for their reduction or cancellation? These are some questions that the chapter means to answer.

Chapter 4 takes up the evoked potentials (137 pp, 51 figures, 181 references, 36 problems); quite a piece, indeed, calling for solid mathematical knowledge, if the reader really aims at grasping the subject. These potentials constitute an event-related activity which occurs as the electrical response from the brain to various types of sensory stimulation. Since interference appears as the main troublemaker, a good portion of the chapter is devoted to how this culprit should be treated. As far as the EP is concerned, noise is essentially synonymous with the spontaneous background EEG activity; however, non-cerebral noise sources must also be taken into account. Ensemble averaging is an important family of noise reduction techniques based on the observation that a stimulus causes a brain response time-synchronized to it. The recorded EEG signal can be transformed into an ensemble of *M *different potentials, with each potential described by *N *samples. Within this family, the chapter describes in detail averaging of homogeneous ensembles, ensemble averaging interpreted as linear filtering, exponential averaging, averaging of inhomogeneous ensembles, spike averaging and robust averaging, effect of latency shifts (even though latencies are many times considered as fixed, variations may occur which introduce distortion), estimation of latency shifts (using, for example, deconvolution), and weighting of averaged evoked potentials using ensemble correlation. Furthermore, the chapter discusses noise reduction by linear filtering, single trial analysis using basis orthonormal functions (a subsection that will certainly impose on the reader's intellectual abilities); thereafter, adaptive analysis using basis functions, and finally, wavelets (32 dense pages) concluding that this transform has been found useful not only for signal denoising but for analysis and characterization of EPs.

The electromyogam is the main actor of chapter 5 (62 pp, 22 figures, 111 references, 14 problems). A good mate to complement it is the second of the books herein reviewed, which, by the way, is the first mentioned by Leif Sörnmo and Pablo Laguna in the list of references. After introducing the signal, these authors consider the amplitude of the EMG as a fundamental quantity which roughly increases monotonically with the force developed by the muscle. However, things are not as straightforward as the previous statement may lead to believe. Since the EMG represents a stochastic event, its amplitude is given by the standard deviation of the observed signal or by a similar dispersion estimate. Thereafter, a couple of pages deal with spectral analysis of the surface EMG, continuing with conduction velocity estimations, modeling of intramuscular EMG and intramuscular EMG signal decomposition. The latter contains important information concerning the motor control system not immediately quantifiable from the measured signal, but which needs to be disentangled with advanced processing and pattern recognition.

The last three chapters take care of the ECG (thus, amounting to about 35% of the book), with chapter 6 (38 pp, 20 figures, 55 references) offering background general information, of the type presented in any physiology text, chapter 7 (91 pp, 44 figures, 204 references, 26 problems, the longest of the three) specifically devoted to the ECG processing, and chapter 8 (54 pp, 24 figures, 98 references, 13 problems) dealing with heart rate variability. Electrocardiographic analysis was one of the very first areas in medicine where computers were introduced. Well respected scientists like Pipberger and Caceres started out the field in the late 1950's and early 60's. So far, however, and in spite of more than 40 years of development and experience, no system offers a universal standard form of ECG signal analysis. In chapter 7, six main subjects are considered in detail: the problem of baseline wander, which tends to modify beat morphology, the ever present headache of powerline interference, interference from muscular activity, the detection of the QRS complex (which may show a large number of morphologies), the problem of wave delineation in order to compute wave durations, and finally, data compression to allow handling of large amounts of information. It is, no doubt, an important chapter requiring from the potential reader good mathematical knowledge and quite a bit of effort, if full profit is to be obtained. The final chapter 8, as already anticipated above, delves into the widespread interest of the non-constant cardiac frequency seen in all normal individuals, including mammals at large. As demonstrated many years ago, in the early 1960's or before (see [3], for more references), a constant heart rate means irreversible damage at the level of the central nervous system, leading to an almost sure death prognosis. I recall Prof. Carlos Vallbona's lectures, at Baylor College of Medicine, in Houston, back in 1966, showing records from patients where such sad event was demonstrated. This chapter discusses acquisition and RR interval conditioning, time domain measures, heart rhythm representations, spectral analysis of heart rate variations (HRV), clustering of beat morphologies, dives into the troublesome problem of dealing with ectopics (their variety and occasional disguised shape makes them a slippery ground). To close the chapter, interaction with other physiological events is treated, as it refers to the effects of respiration and blood pressure [4].

The book carries also two appendices: One reviews important concepts needed for better understanding the tools applied in its contents, such as matrix fundamentals and discrete time stochastic processes. The other, in particular, I welcome warmly because it lacks in other books and has been my tired complain; it lists symbols and abbreviations. Good idea! Sometimes authors think the acronym is so obvious that everybody should know ... and the truth is that not everybody knows!

A nice and not frequent feature is the existence of two websites, one supplies the solutions to all the proposed problems and the other holds several project descriptions and offers signals available for download. Bibliography is abundant (just take a look at its number) and anyone who wants to deepen in his/her subject of attraction will find the lead to proceed further. Reference [5] might be a good companion for this book, too.

Now, let us get into the second book starting with a musing ... *The Muscular System ... we look at it almost with worship, it is the driving engine to move us about and beyond, to fight and run; it gives us joy and pleasure, and yet, how far behind the mind it is *[2].

The dream told by Mary Wollaston Shelley in her Frankeinstein story back in 1815–6, as the first science fiction novel, has been more than surpassed by the world we live in. Biomedical Engineering, no doubt, has played a significant role in such development and the book we are about to comment appears as an excellent sample example of such contributions. Was Luigi Galvani not the discoverer and beginner of animal electricity? Were his frogs not *le prime donne *during the three or four early XIXth Century decades, even in social gatherings, to show how the *quasi-dead *could be made to move or to indicate the presence of an action potential with the *rheoscopic preparation *in research labs? Was Frankeinstein's monster not created by electrical discharges through appropriate electrodes? The electrical signal generated by skeletal muscles signaled the birth of electrophysiology, direct current electricity (after the controversy with Alessandro Volta) and the literary science fiction style! Let me offer this comment and through it, with Roberto's and Philip's kind permission (even though I have not asked specifically for it), this book as silent respectful homage to that dedicated Italian obstetrician and true researcher who carried out his breakthrough experiments in his Bologna house, using rudimentary technology and tremendous ingenuity to design, manual skill to actually do it and genius to interpret, helped by his wife, often going through economic distress [2,6]. Thus, **hail to Luigi Galvani, founder of the EMG!**

The editors of this solid book belong, respectively, to the Politecnico di Torino, Italy, and to the University of New Brunswick, Canada. They have ample and recognized experience in the subject. The book, in turn, is sponsored by the IEEE Press Engineering in Medicine and Biology Society, being part of the Series in Biomedical Engineering, with Metin Akay as Series Editor. Technical reviewers were Paolo Bonato, from Harvard Medical School, and Guruprasad Madhavan, from State University of New York. All this, no doubt, represents more than enough guarantee seal of top quality. Besides, the list of contributors (33, all hard scientists and professionals from outstanding institutions) is impressive because of its average academic high level. Sweating task for an oldie modest researcher pretending to critically review it!

In the introduction (4 pp, 44 references), the Editors give a short historical note and general overview of the book. Nice beginning. Quoting almost verbatim from them, the book is dedicated to the EMG, the signal generated by skeletal muscles; motors that allow us to move about. Piper is considered to be the first investigator to study EMG signals. He did it in Germany in 1912 using a string galvanometer. In 1924, Gasser and Erlanger carried out similar investigations with an oscilloscope. Remember they won the Nobel Prize for their contributions in nerve conduction. From there on, smaller and bigger discoveries and additions began to pile up getting to the current status, where needle and surface EMG techniques are complementary instruments and integrate each other. The book is aimed at graduate students in biomedical engineering, life sciences, movement sciences, exercise physiology, neurophysiology and neurology, rehabilitation, sports, occupational medicine, and related fields. A background in biomedical instrumentation, signal processing and mathematical modeling is assumed.

The first chapter (20 pp, 13 figures, 105 references), by T. Moritani, D. Stegeman and R. Merletti (who co-authored 6 and, thus, appears as the main leader), is a brief refreshing of the physiology and biophysics of the muscular electrical signal. Those already versed in it can skip it and those who want to improve their knowledge will find enough referred literature to satisfy their needs. Chapter 2 (19 pp, 13 figures, 26 references), written by J.V. Trontelj, J. Jabre and M. Mihelin, continues within the introductory trend describing needle and wire detection techniques. *Nihil novum sub solem*, but it is illustrative. D.W. Stashuk, D. Farina and K. Søgaard, authors of the third chapter (31 pp, 17 figures, 68 references), get more into the main subject by taking up the decomposition of intramuscular signals. Monitoring of motor units (MU) recruitment, derecruitment, and firing rate, leads to the understanding of motor control strategies and of their pathological alterations. EMG decomposition is the process of identification and classification of individual MU action potentials in the interference pattern detected with either intramuscular or surface electrodes. The task is complex for it involves advanced signal processing and pattern recognition techniques using, for example, the so called *clustering algorithms*. As pointed out by the chapter's authors, however, EMG signal decomposition is still carried out mainly in research environments while it finds limited clinical application.

Chapter 4 takes up the biophysics of the EMG signal generation (22 pp, 11 figures, 73 references). It was written by D. Farina, R. Merletti and D.F. Stegeman; they describe basic concepts of generation and detection of the electrical muscular event paying attention to the appearance at the end-plate, propagation along the sarcolemma, and extinction at tendons, crosstalk between nearby muscles, selectivity of the detection system in relation to the signal sources and the volume conductor properties. Lastly, the relationship between the developed force and the characteristics of the surface EMG is presented. There is some degree of overlapping with the first chapter.

Technical aspects become the concern of chapter 5 (21 pp, 10 figures, 4 tables, 67 references), authored by R. Merletti and H.J. Hermens. Its objective tries to clarify concepts regarding the electrode size, inter-electrodic distance, electrode configuration and location as well as issues related to the input amplifier. Besides, filter specifications, sampling, A/D conversion and general European recommendations are also given. It is underlined that standardization efforts always lag behind research and are never completed; I would add that they evolve and need continuing revision and update.

The trio formed by E.A. Clancy (from Worcester, MA, USA), D. Farina (from Torino, Italy) and G. Filligoi (from La Sapienza, Rome, Italy) was in charge of chapter 6 (31 pp, 10 figures, 114 references), titled "single-channel techniques for information extraction from the surface EMG signal". They are used to study the interference pattern that results from the simultaneous activation of many motor units. These techniques do not resolve the signal into individual MUs, rather they provide a global description of the electric potential observed at the recording site focusing on the relationships with the underlying physiological processes. The following chapter 7 (31 pp, 17 figures, 88 references), by Farina, Merletti and Disselhorst-Klug, constitutes with the former a small package because it deals with multichannel techniques, thus complementing each other. The surface EMG presents a smaller bandwidth with respect to the intra-muscular signal since the tissues between the muscle fibers and the electrodes act as low-pass filters leading to low spatial selectivity, which hinders the separation of the motor unit contributions. The chapter discusses spatial filtering and sampling and muscle fiber conduction velocity, all within an excellent mathematical framework. The authors add also that the extraction of single MU action potential trains from surface EMG remains a difficult task only to be performed under particular conditions.

Up to here, we reach roughly to about 42–43% of the book (introduction and 7 chapters, 205 pp, without counting the index pages); the remaining (11 chapters, 270 pp) is devoted to more special subjects, which might better call the attention of the specialist, not meaning that they lack interest, quite the opposite, for they show new avenues for discovery, invention and advancement.

The abovementioned series begins with chapter 8 (23 pp, 11 figures, 80 references), authored by D.F. Stegeman, R. Merletti and H.J. Hermens, and gets into modeling and simulation of the EMG. The authors say in the conclusions that, since the mid-1970's, a considerable number of papers on the use and development of EMG models were published. Models are particularly helpful in obtaining insight into the basic elements of surface and needle EMG characteristics. By and large, what is involved is the search for a uniform approach. Considering the variety of questions, such a goal will probably never be reached in full. The present overview is meant to give some background and suggests the possibilities of surface EMG and their limitations.

Muscle fatigue belongs to the daily experience of everybody and has been the concern of physiologists for centuries. There is a proprioceptive feeling which in the end becomes painful, there are metabolic and biochemical changes, the actomyosin machinery stops responding as it should, training is a major factor in extending its appearance but ... how can it be unequivocally defined? How can it be quantified? What are the myoelectric manifestations? These are the questions addressed to in chapter 9 (21 pp, 12 figures, 1 table, 93 references), by R. Merletti, A. Rainoldi and D. Farina; attractive subject and full of possibilities, no doubt.

**Figure 1:**
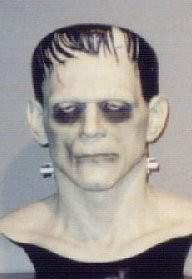
Frankenstein's Monster bust, displayed at the Bakken Museum and Library, Minneapolis, MN, USA. The picture was taken by the author of this review as fellow of that institution in September 2002.

Those interested in going deeper into the processing techniques should take advantage of chapter 10 (44 pp, 18 figures, 56 references), written by D. Zazula, S. Karlsson and C. Doncardi), however, and with due respect to the chapter's authors and the editors and without entering into its contents (which is definitely good), this chapter perhaps could have been spared because the available literature is enormous and so making the book a little shorter. A few specific applications (as in EMG decomposition or for studying muscular fatigue) could have been inserted in the sections devoted to those subjects.

Prof. C. Orizio, from the University of Brescia, Italy, starts out his chapter on the surface mechanomyogram (the 11^th ^of the book, 14 pp, 10 figures, 71 references), with the following nice paragraph: "Even during muscular isometric contraction, when the muscle tendon unit is kept at constant length, the sliding of the actomyosin filaments determines a reduction of the length of the contractile elements of the muscle with a shortening of its long axis. Since muscle can be considered as a near constant volume system, these changes in muscle length are paralleled by changes in the transverse axis dimension. Indeed, already at the end of the nineteenth century the French physiologist Marey designed specific myographs to record the changes in the muscle thickness during evoked contraction. Analysis of the transverse diameter changes of muscle was considered as a reliable tool and since the beginning of the twentieth century was used to describe the muscle contraction process in several handbooks of physiology." Subtitles of the chapter refer to general aspects of the mechanomyogram (MMG) during stimulated and voluntary contraction, detection techniques and sensors comparison (laser, accelerometers, piezoelectric elements, microphones), simulation, MMG and force, MMG and EMG. Good condensed chapter, good bibliography and an author collecting good experience. *Ben fatto e diretto al ponto, dottore Orizio, mi ha piaciutto*! Warning: The abbreviation MMG stands also in the literature for magnetomyogram, thus, be careful.

We reach another short piece full of information, chapter 12 (19 pp, 15 figures, 26 references), this time dealing with applications in neurology of the surface EMG (sEMG), by M.J. Zwarts, D.F. Stegeman and J.G. van Dijk, all them from The Netherlands. Among the several studies a neurologist may order a patient undergoing pathologies of this kind, nerve conduction tests and EMG are frequently used. Essentially two approaches are available based on sEMG: polymyography (to evaluate many muscles) and/or eliciting some kind of reflex. Compound muscle potentials and motor nerve conduction are commonly required along with the so called muscle cartography; the latter finds a place to infer the number of functional motor units. New developments are the application of high-density multichannel grids that enable non-invasive measurement of motor units' characteristics such as firing behavior, position, and sarcolemmal properties.

Ergonomics is not a frequent subject and I particularly welcome its inclusion as chapter 13 (16 pp, 4 figures, 103 references), in charge of G.M. Hägg, B. Melin and R. Kadefors, all from Sweden. My only complain concerns its length: too short, leaving the reader wishing for more. No doubt, as stated by the authors, physical load represents an important aspect in the design of work and workplaces, especially when the strain localizes on certain parts of the musculoskeletal system. After touching briefly on several topics, the chapter concludes that several experimental studies have confirmed that psychological stress or cognitive factors, even in the absence of physical demands, can increase muscle tension as reflected by the EMG.

Chapters 14, 15 and 16 comprise well related areas and, therefore, it appears as acceptable to comment them as a set: exercise physiology (13 pp, 5 figures, 44 references, by F. Felici), movement and gait analysis (17 pp, 10 figures, 74 references, by C. Frigo and R. Schiavi), and rehabilitation medicine (22 pp, 8 figures, 153 references), by A. Rainoldi, R. Casale, O. Hodges and G. Jull), respectively. Strength, power training and muscle damage are salient topics of the first of these chapters that unfortunately are too scarce in content. The second of the series discusses the relevance of EMG in kinesiology, the acquisition settings, the motor control strategies, not very much on gait analysis (even though it is part of the chapter's title), the identification of pathophysiological factors, workload assessment in occupational biomechanics, the use of the linear envelope in representing EMG patterns, and multifactorial analysis. The last one aims at providing an overview of the literature in frontier fields where surface EMG may have a potentially significant role and outline a few existing applications, such as back and neck pain, pathologies of the pelvic floor, age-related effects of muscle physiology, hypobaric hypoxia and microgravity.

The end of the book is getting close. Prof. J.R. Cram, from Nevada City, CA, USA, wrote about feedback applications (chapter 17, 16 pp, 9 figures, 2 tables, 20 references). It intends to tell how surface EMG may be used in a clinical setting as biofeedback tool, which find a place in psychology and physical medicine. Subjects discussed include psychophysiological stress-related hyperactivity, postural dysfunction, weakness/deconditioning syndrome, acute reflexive spasm/inhibition, learned guarding/bracing, learned inhibition/weakness compensation for joint hypermobility or hypomobility, chronic faulty motor programs and a set of surface EMG biofeedback techniques.

A great area full of possibilities is that of powered upper limb prostheses. Unfortunately, wars, terrorist attacks and car and motorcycle accidents are filling endless lists of amputees, many very young who, otherwise, have an alert and motivated mind. This final chapter 18 (19 pp, 8 figures, 81 references), authored by P.A. Parker, K.B. Englehart and B.S. Hudgins, all from New Brunswick, Canada, deals precisely with this subject. The myoelectric signal serves as an important control input for powered prostheses. Such signal conveys information regarding intent from the user to the prosthesis controller. The control information can vary from simple switch on-off commands to complex multifunction commands. The biomedical engineer faces a tremendous modeling task to improve the overall understanding of these inter-related systems, with non-linear mathematics and control systems as essential tools. Artificial limbs or prostheses of different kind appear as challenging aids to the amputee. Such devices precisely intend to replace one part of the musculoskeletal system (a hand, an arm, a leg). One of them is the so-called *myoelectric limb*, in which the control signal is derived from surface electrodes picking up nerve or muscle action potentials still under voluntary control of the patient. The signal is processed by electronic circuitry to reach a decision as to whether a function is to be activated, usually a predetermined movement of the artificial limb. However, for the signal to be of clinical value in controlling a prosthesis, an estimation process is required that must not take much more than one tenth of a second, otherwise, an unacceptable delay will occur between the amputees' contraction and the action of the prosthetic. The task facing the amputee in using myoelectric prosthesis is difficult. It involves generating a myoelectric signal level corresponding to a specific target. The difference between the signal actually generated and what was intended is termed "operator's error". Thus, the actual behavior of the myoelectric prosthesis results from the intent of the amputee modified by two sources of error, the system error and the operator's error. Neither of them can be eliminated. The subject is quite a challenge for the bioengineer, for the physician and, above all, for the patient, who also generates the need of psychological support studies.

Well, the whole book was browsed over. I recommend it without doubt and would like to add a few minor comments to finish up this review:

The index (18 pp) is good, goes into enough detail to rapidly find a given subject avoiding unnecessary lengthy lists of words and subdivisions that sometimes tend to confuse the reader.

Some authors seem to have wanted to compensate a lean content with far too many references, as if implicitly telling the reader to search further following the leads given in the text. Well, such a philosophy sounds fine ... up to certain limit not easy to establish because it is a question of personal criterion. Overall, the book could have deleted one or two chapters, merge two or three, and deepen the contents of a few.

As usual nowadays with many books, acronyms abound, and I miss an appendix listing their definitions, even though they may appear interspersed in the text. My frequent complain refers to the forgetfulness or neglect for the poor guy who is not aware or enough knowledgeable of the subject matter. Oh, those sentences full of abbreviations, like unfriendly porcupines or barbed wire ... just for the sake of shortness! In the end, the suffering reader spends more time searching for their meaning ... Please, dear authors, don't take this wrongly, simply, understand me, it means to be a constructive and friendly criticism. Well, I always complain because my ideal is the unreachable perfection, which is only in the hands of the Great Engineer!

As mentioned early above, the two books are subject related and, thus, their reviews were experimentally merged in one, as a novel approach to this category of article. References [7-9] are perhaps pertinent to complement information. Besides, to give the review an entertaining touch, let me illustrate with Figure 1; it is a Frankenstein's Monster bust, displayed at the Bakken Museum and Library, Minneapolis, MN, USA.
